# Do morphological and physiological characteristics of males of the dragonfly Macrothemis imitans determine the winner of territorial contests?

**DOI:** 10.1093/jis/14.1.89

**Published:** 2014-07-08

**Authors:** M. A. N. Mourão, P. E. C. Peixoto

**Affiliations:** 1 Programa de Pós-Graduação em Ecologia e Recursos Naturais, Universidade Federal do Ceará, Ceará, Brazil; 2 Instituto Brasileiro do Meio Ambiente e dos Recursos Naturais Renováveis, Brasília CEP 70818-900, Distrito Federal, Brazil; 3 Departamento de Ciências Biológicas, Universidade Estadual de Feira de Santana, Feira de Santana CEP 44036-900, Bahia, Brazil

**Keywords:** sexual selection, territoriality, intra-sexual selection, agonistic interactions

## Abstract

Males of many animal species show intraspecific disputes for mating territories that range from displays without physical contact to physical fights with risk of injury. This variation motivated the proposition of different models that suggest possible rules used by rivals to decide the contest winner. To evaluate those models, it is necessary to identify how males behave during the fight and the individual attributes that determine their fighting ability (resource holding potential). For this, males of the dragonfly *Macrothemis imitans* (Karsch) (Odonata: Libellulidae) were used to evaluate two hypotheses conditioned on the occurrence of physical contact during the fight: if the contests occur with physical contact, features related to size should determine male resource holding potential, and if males do not exhibit physical contact during the contests, features that confer greater endurance should determine resource holding potential. To assess these hypotheses, we collected males that had ownership of territories (resident males) and males that occupied the territory after we removed the resident males (substitute males). After the capture, the resident and substitute males were transferred to the laboratory for measurements of wing area, dry weight, thoracic muscle mass, and fat content. The results showed that resident males do not differ in any measured trait from substitutes. Because the fights occur with physical contact, it is intriguing that resident males do not possess higher fighting capacity than intruders. Perhaps physical contact does not incur high costs during the fight, and other asymmetries, such as motivation associated with prior residency of the disputed territory, determine the contest winner.

## Introduction

In many animal species, males fight for ownership of territories for mating purposes ( Andersson 1994 ). The fighting behaviors used by males differ greatly among species ( Arnott and El wood 2009 ), varying from disputes without any contact between the rivals (e.g., Alcock and Bailey 1997 ) to interactions with physical contact, injuries, and risk of death (e.g., Bean and Cook 2001 ). Such variation is common even in species that fight for the possession of mating sites during flight, such as butterflies, dragonflies, and damselflies ( Marden and Waage 1990 , Peixoto and Benson 2011a , Junior and Peixoto 2013 ).

Many theoretical models aim to explain the rules used by males to decide the winner of a territorial contest ( Enquist and Leimar 1983 , Mesterton-Gibbons et al. 1996 , Payne 1998 ). Despite differences among these models about the ways by which disputes are decided, all assume that the chance of winning depends on two parameters: (1) the individual’s fighting ability (RHP, resource holding potential; Parker 1974 ), which can be determined by physical (e.g., size and weight) or physiological traits (e.g., fat, muscle, and immunocompetence) (see Suhonen et al. 2008 ); and (2) the individual’s motivation to invest in the contest (RV, resource value; Parker 1974 ), which is often determined by the previous experience of the owner of the contested resource and by the local quality of the defended site (e.g., Briffa and Elwood 2001 , Bergman et al. 2010 ).

Because all the models previously mentioned postulate that RHP differences between rivals determine the chance of winning, to be able to evaluate such models empirically it is necessary to identify which morphological or physiological traits determine the males’ most often used for this purpose is to establish correlations between specific male traits and the chances of winning a territorial contest. However, this approach is unsuitable in establishing the mechanism that connects the trait to how costs are accrued during the contest, and it can lead to the establishment of spurious relationships ( Lailvaux and Irschick 2006 ).

To avoid these results, it is necessary to establish the functional relationship between male traits and fighting behavior ( Irschick et al. 2007 ). One way to establish this functional relationship is to classify the fighting behaviors according to the occurrence of physical contact between rivals (e.g., Peixoto and Benson 2011b ). If the fights occur with physical contact, injuries should be the main form of cost accrual during the fight. In this sense, features such as body mass should increase the individual’s resistance to injuries from rivals or the ability to inflict damage. Therefore, traits related to physical strength must be functionally important to determine male RHP (e.g., Forslund 2000 ). If fights occur without physical contact, however, the costs should be determined by the energy depletion during the fight. The winner must be the individual that is able to persist for a longer period in the contest. Consequently, physiological features such as energetic reserves may be more important in determining the individual’s fighting ability (e.g., Marden and Waage 1990 ).

In many dragonfly species, males establish mating territories and fight for the ownership of these sites ( Corbet 1980 , Córdoba-Aguilar 2008 ). The fighting behaviors usually comprise a combination of circular chase flights, upward spiral flights, and straight back-and-forth persecutions ( Córdoba-Aguilar 2008 ). In some species, males may exhibit physical contact during disputes ( Corbet 1999 , Switzer and Schultz 2000 , Junior and Peixoto 2013 ), but in others, the males exhibit displays without contact (Waage 1988, Switzer 2004 ). Features such as energy reserves ( Marden and Rollins 1994 ) and parasitism ( Contreras-Garduño et al. 2006 ) seem to predominate as RHP determinants in this group.

Males of the dragonfly *Macrothemis imitans* (Karsch) (Odonata: Libellulidae) are found along creeks and stream banks, where they presumably roam in search for females among patches with macrophytes or, in most cases, among exposed rocks and sandbars (personal observation). Territorial fights and matings are frequently observed. Despite the high occurrence of agonistic interactions, however, it is unknown what behaviors are used by rivals during the dispute and what characteristics determine male RHP. Therefore, in this work we proposed to evaluate whether physical contact is a common property of the territorial disputes and to identify which morphological or physiological traits determine the winning chances of male *M. imitans* in disputes for territory ownership. If contests occur with physical contact, we hypothesize that features related to size will determine male RHP. Alternatively, if males do not exhibit physical contact during the contests, we hypothesize that features that confer greater capacity to remain in flight will determine male RHP.

## Materials and Methods

### Study area

The study was conducted in a 130-m stretch of Urubú stream (15°42'S, 47°51'W), located about 9.5 km from the center of Brasilia, DF, Brazil. The average depth of the stream at that location was 60 cm, and the average width was 3.5 m. The weather is tropical-high altitude, with two distinct seasons: a wet and rainy summer, with an average temperature of 29.7°C (SD = 0.72) and average rainfall of 203.9 mm (SD = 27.2), and a dry and cold winter with an average temperature of 12.5°C (SD = 2.5) and average rainfall of 24.9 mm (SD = 76.7) (Inmet 2011).

### Description of territorial behavior

To evaluate whether disputes occur with or without physical contact, samples were collected in campaigns composed of two consecutive days between November 2011 and March 2012. On the first day of each campaign, several males were marked with a number written on one wing with a black permanent marker that allowed visual identification of males without recapture. On the second day, behavioral observations of males and the removal experiment were done to evaluate the traits associated with the chance of winning a contest (see “Removal experiments” below for details). Behavioral observations were made on 31 resident males (males that were defending the territories at the beginning of our observations) and 31 substitute males (males that occupied the territories after the resident males were experimentally removed) only on sunny days from 09:00 to 14:00, when individuals are more active. During the observations, the behaviors of individuals were recorded for 10 min using a mini-portable recorder, with particular reference to the occurrence of physical contact during territorial disputes exhibited by the observed males ( Table 1 ). Afterwards, the recordings were used to time how long each individual spent in each behavior.

**Table 1.**
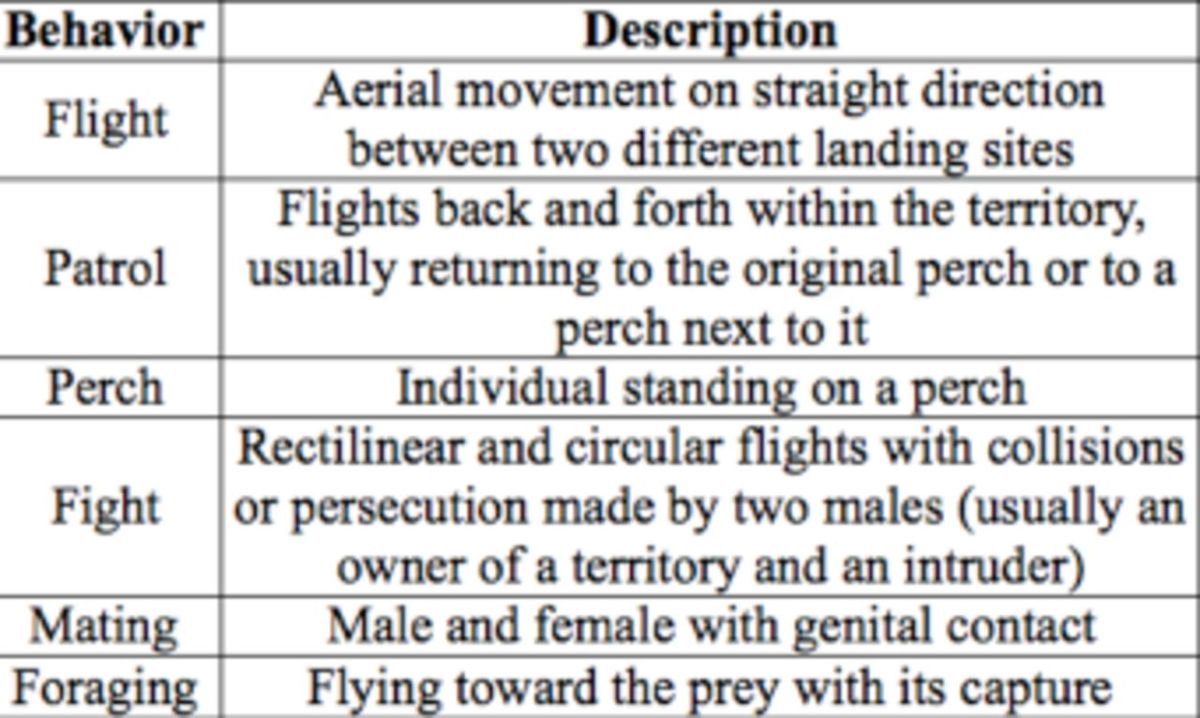
Descriptions of the behavioral categories exhibited by male *Macrothemis imitans* during observations.

### Removal experiment

After the behavioral observation, we performed a removal experiment to evaluate male traits that may determine the winning chances. For this experiment, whenever a male exhibited signals of territorial defense (i.e., fights with conspecific males or flight patrols), it was removed from the defended site (resident male). After the male was removed, the territory remained under observation until a new male arrived (substitute male). Upon arriving on the observed site, the substitute male was captured to receive a mark, consisting of a point on one wing. This approach was done with only one male at a time, and it never took more than 1 min to avoid stress on the manipulated animal (all marked males resumed the territorial defense after being released). After releasing the marked substitute male, it was observed for 10 min and then captured. After this, both males (resident and substitute) were measured for weight, wing area, fat content, and residual muscle mass (for a detailed description, see “Morphological and physiological measures” below).

Removal experiments have been used successfully to evaluate male traits that are important for contest resolution ( Peixoto and Benson 2008, 2011a ). In these experiments, it is expected that resident males are individuals that have disputed the ownership of territories and won, whereas substitute males represent weaker individuals that were unable to obtain territories. Thus, if fights occur with physical contact, it is expected that resident males have a greater wing area or greater body mass than the rival males. Alternatively, if fights occur without physical contact, it is expected that resident males have a greater fat content or greater investment in thoracic muscles for flight than substitute males.

### Morphological and physiological measures

A caliper was used to measure the length and width of each wing. The wing length was measured as the distance between the wing insertion on the thorax and its apex. The width was recorded as the longest distance between the wing edges on the perpendicular direction to the length axis. Wing area was estimated by calculating the ellipse area, considering the wing length as the largest diameter and width as the smallest diameter. Then, the wing area of the four wings was added to estimate the total wing area for each male.

To estimate fat content, males were kept in an oven at ∽60°C for 48 hr. Later, the thorax (without wings and legs) and abdomen were separately weighed on a balance (accuracy 0.0001 g). The summed mass of thorax and abdomen after dehydration was used as a measure of male size (dry mass). After weighing, both parts of each animal were submerged in a closed bottle containing 10 mL of chloroform for 48 hr to extract lipids. After removing them from the chloroform, the body parts were kept in an oven at 60°C for an additional 48 hr before being weighed again. To estimate the amount of thoracic muscle mass available for flight (flight muscle ratio), the same procedure was used. However, only the thorax weight (after lipid extraction) was measured before and after being immersed in 0.2 M potassium hydroxide ( Plaistow and Siva-Jothy 1999 ). The mass difference before and after immersion in chloroform was used to estimate fat content, and the mass difference before and after the immersion in potassium hydroxide was used to estimate muscle mass. Because larger individuals tend to have more fat and muscle mass than smaller ones, we calculated the residuals of a linear regression between the fat content and the dry body mass of individuals before the lipid extraction (residual fat) and between the muscle mass and dry body mass (residual muscle mass) to obtain values of fat content and muscle mass independent of size ( Marden 1989 , Marden and Chai 1991 , Marden and Rollins 1994 ).

### Statistical analysis

A logistic regression was used to assess the effect of each trait (wing area, dry weight, residual fat content, and residual muscle mass) on the probability of a focal male to be territorial ( Hosmer and Lemeshow 1989 ). Because data were collected from pairs of males (resident and substitute), we adopted a modification to maintain the pairing (Kemp 2000). For this, 16 pairs were randomly chosen. For each pair, the resident individual was designated as the focal male (focal state 1). For the remaining 15 pairs, the substitute male was designated as focal individual (focal state 0). When the focal state was 1, the values of resident male traits were subtracted from the values of its substitute pair. Inversely, when the focal state was 0, the values of the substitute male were subtracted from the values of its resident pair. Thus, if resident males possess a higher fighting ability than substitutes, it was expected to find positive values for the differences between male traits associated with focal pairs whose state was 1 and negative values associated with focal pairs whose state was 0 (e.g., Peixoto and Benson 2008 ). To identify the most parsimonious model describing the relationship between the focal status and differences in the male traits, we used a corrected version for small samples of the Akaike information criterion ( Burnham and Anderson 2002 ).

## Results

### Description of territorial behavior

**Figure 1.**
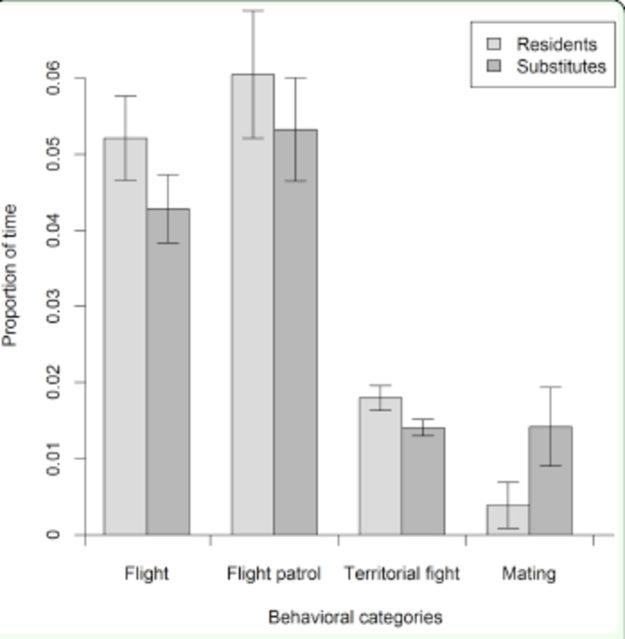
Average proportion of time that the resident and substitute males of the dragonfly *Macrothemis imitans* spent in each behavior during 10 min of behavioral observation. The proportion of time in which males remained resting is not shown. Bars represent the standard error. High quality figures are available online.

Resident and substitute males showed a very similar behavioral pattern ( Fig. 1 ). Resident and substitute males stayed perched for the greatest proportion of the time (resident males: mean = 519.2 s, SD = 41.1; substitute males: mean = 524.8 s, SD = 39.2). When males were not perched, they spent most of their time in flight and flight patrol, respectively. Although the 62 males (resident and substitute combined) had been involved in 180 disputes, the proportion of time spent in fights accounted for a smaller proportion of the time budget of males. On average, the fights lasted 10.1 sec (SD = 5.3) for resident males and 8.09 sec (SD = 3.88) for substitute males.

Fights between males always initiated when a male entered the site defended by a resident or by a substitute male. In both situations, the territory owners flew toward the intruders, colliding against them. After the collision, the territory owner (for both resident and substitute males) performed straight flights and/or circular chase or collision against the intruder. After expelling the invader, the winner returned to the perch of origin. The resident males were involved in 99 disputes and won 100% of the contests, and the substitute males were involved in 81 disputes and also won 100% of the contests. We observed two matings performed by resident males and eight matings by substitute males (corresponding to the smaller proportion of the time budget of males). No individual without a territory was observed mating.

### Removal Experiment

**Figure 2.**
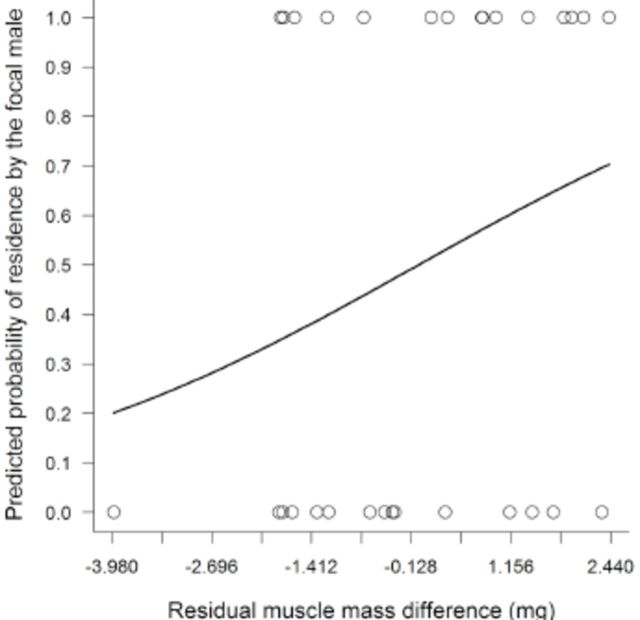
Probability of the focal male *Macrothemis imitans* to be the resident in relation the residual muscle mass difference from its non-focal pair. Overlapping points represent different samples with the same focal result and residual muscle mass difference. High quality figures are available online.

In relationship to the individual traits that may determine the chance of the focal male being the resident, the model that contains only residual muscle mass difference was selected as the most parsimonious candidate ( Table 2 ). However, this model indicates that the residual muscle mass is unimportant in determining the chances of being the resident male ( Table 2 ; Fig. 2 ).

**Table 2.**
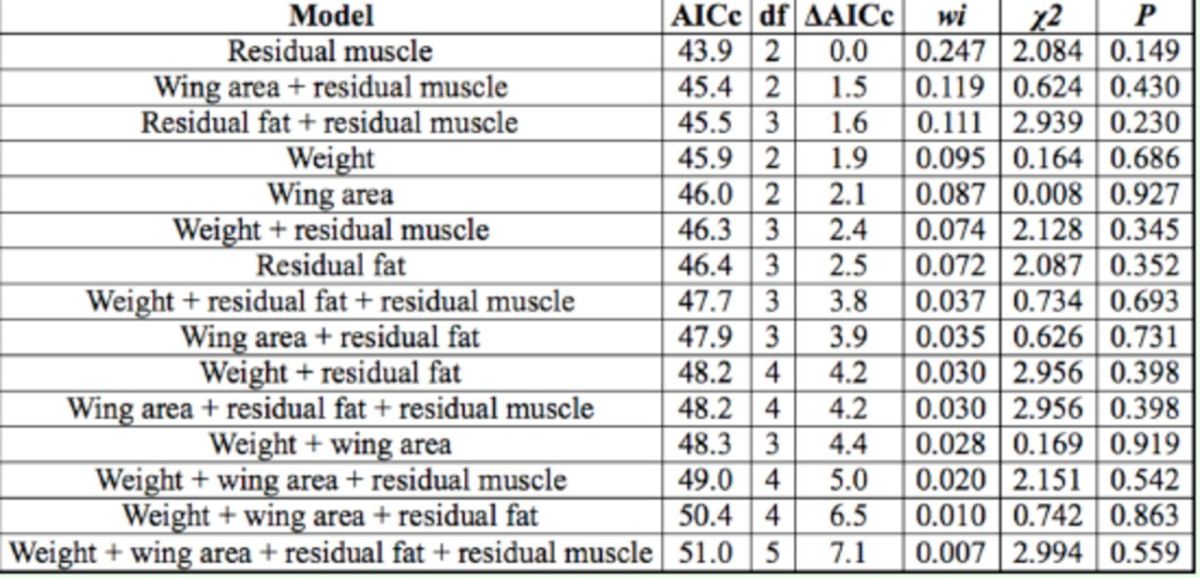
Summary of the logistic model describing the probability of the focal male of Macrothemis imitans (n = 31) being the resident in relationship to the value difference between their traits and its non-focal pair.

The models are ordered according to their AICc values (AICc represents the value of the Akaike Information Criterion corrected for small samples; Äi is the value difference between the most parsimonious model and model i, wi is the weight of Akaike model i).

## Discussion

Territorial males of *M. imitans* (residents and substitutes) remained most of the time perched. The fact that the time budget of substitute males was similar to that of the residents indicates that the marking manipulation did not affect their behavior. Because the fights accounted for a small part of their time budget, it is possible that the energy costs accrued during continuous flight in the contests are low. The fact that there are collisions between males during the fights, however, indicates that injuries may represent costs. In this sense, it was expected that features related to size should determine male RHP ( Lailvaux and Irschick 2006 ). Contrary to expectations, the chance of a male to be the resident individual was unrelated to its morphological or physiological traits. Perhaps the removal of resident males a short time after the beginning of territorial activity has reduced their chance to be the individuals that won the fights, which could obscure eventual differences between winners and losers in determining residency. Nevertheless, it is common among territorial insects that the resident males are the first to arrive in the territory, and they stay defending the same place on consecutive days (e.g., Forsyth and Montgomerie 1987 , Peixoto and Benson 2009 ). The fact that the resident male won 100% of the disputes indicates that it was the individual with greater winning chances. Consequently, it is improbable that the resident males were wrongly identified during the removal experiment.

Because resident and substitute males in ownership of the territories won all the fights, it is possible that the asymmetry in territorial status determines the winner ( Davies 1978 ). Although there is a lack of clear empirical demonstrations, theoretical models suggest that males may determine the contest winner through uncorrelated asymmetries, such as the simple distinction between territory owners and intruders ( Maynard Smith and Parker 1976 , Kokko et al. 2006 ). It is also possible that disputes are settled on the basis of differences in territory value for each rival ( Parker and Rubenstein 1981 , Enquist and Leimar 1987 ). In many species, individuals in ownership of territories present greater investment in fights than intruder rivals ( Switzer 2004 , Takeuchi and Honda 2009 , Bergman et al. 2010 , Peixoto and Benson 2011b ). Consequently, it is possible that even individuals with lower fighting ability remain as residents because asymmetries in motivation associated with the value that each rival attributes to the territory.

The lack of correspondence between male traits that are important in determining residency and the occurrence of physical contact between rivals during the fights is intriguing. It is possible that injuries are, in fact, an important fighting cost (e.g., Junior and Peixoto 2013 ). In this situation, there may be alternative individual traits that affect contest settlement. Perhaps the fighting ability of males is not determined solely by a particular trait, but by a combination of different characteristics that determine the overall male condition ( Kemp et al. 2006 ). On the other hand, Vieira and Peixoto (2013) have demonstrated that the functional relationship between fighting behaviors and how individual traits affect cost accrual during the fight is not universal. In this sense, it is possible that collisions do not generate any fighting cost. If this is true, disputes should be mainly determined by asymmetries in male motivation associated with differences in resource value, as occur in some butterfly species ( Takeuchi and Honda 2009 , Bergman et al. 2010 ).

## Abbreviations

RHP: resource holding potential
